# Traditionalist, supplementer, or reformer? medical student use of non-official curricular resources

**DOI:** 10.5116/ijme.6a31.72ef

**Published:** 2026-07-03

**Authors:** Donovan Makus, Jessica Maher, Mark Labib, Anushu Kashyap, Susan Humphrey-Murto

**Affiliations:** 1 Department of Medicine (Internal Medicine), University of Calgary, Calgary, Canada; 2 Faculty of Medicine, University of Ottawa, Ottawa, Canada; 3 Department of Family Medicine, McMaster University, Hamilton, Canada; 4 Department of Medicine, University of Ottawa, Ottawa, Canada

**Keywords:** Non-traditional resources, commercial learning platforms, self-regulated learning

## Abstract

**Objectives:**

The purpose of this study was to explore why and how medical students use non-traditional learning resources relative to the formal curriculum, to inform curriculum development efforts, and support self‑directed learning.

**Methods:**

A qualitative study grounded in a pragmatic research approach was conducted using semi structured interviews with medical students at the University of Ottawa. Participant recruitment occurred via email/social media, and a pre survey was used to ensure sampling of both low- and high-level resource users. Transcripts were analyzed using reflexive thematic analysis in NVivo. Analysis was both deductive, guided by self-regulated learning (motivation, goal setting, feedback, self-monitoring), and inductive to capture unanticipated themes.

**Results:**

Twenty-nine students participated (18 pre-clerkship; 11 clerkship). Four themes were developed: two addressing motivations  for using non‑traditional resources—the traditional curriculum is repetitive and inflexible and non‑traditional resources are high‑yield and flexible; one addressing goal setting—studying for today’s exam or tomorrow’s patient; and one addressing how students engage with resources, captured through three archetypes—the Traditionalist, the Supplementer, and the Reformer.

**Conclusions:**

This study demonstrates how medical students navigate learning by turning to non-traditional resources, shaped by their motivations, goal orientations, and distinct engagement patterns. These insights highlight opportunities to streamline and modernize curricula, integrate vetted high yield resources, and strengthen students’ self-regulated learning skills. Leveraging the three learner archetypes can further guide curriculum planning by recognizing diverse learning approaches, engaging Supplementers as indicators of curricular gaps, supporting Traditionalists with structured pathways, and viewing Reformers’ non-attendance as an expression of SRL rather than disengagement.

## Introduction

Non-traditional learning resources—materials not produced by a student’s own institution or lecturers—have become central to medical education. These include peer-generated note decks, spaced repetition platforms, and commercial off-the-shelf learning platforms developed specifically for medical education labelled "MedED-COTS".^1^ MedED-COTS, are widely used: in 2022, 96.2% of American medical students reported using non-traditional resources, with 36.6% using them daily.^2^ Similar patterns have emerged internationally, for example 70% of clerks in the United Kingdom reporting utilization of medical apps such as UpToDate and uWorld.^3^^–^^7^Their use has been linked to higher performance on standardized exams such as the USMLE.^8^ Outside the U.S., however, usage is less well studied, and it remains unclear whether patterns such as reduced class attendance are related to reliance on non-traditional resources.^9^ While a recent Best Evidence Medical Education (BEME) guide endorsed their value, most existing studies remain survey-based, with limited qualitative insight into students’ motivations.^1^

A recent focus group study partially addressed this gap, reporting that pre-clerkship students preferred non-traditional tools for their efficiency, clarity, and peer endorsement.^6^ These findings highlighted the influence of high-stakes national exams like USMLE Step 1 and 2, but the study focused solely on the American context, and relied on focus groups, where disclosure may be constrained by social dynamics.

To date, while usage rates have been well documented,^2^^–^^6^key questions remain about how and why students use these resources. Students appear to be selecting different resources to enable their own learning which fits within the framework of self-regulated learning (SRL). SRL is a cyclical process in which learners set goals, select strategies, monitor progress, and reflect on outcomes.^10^ Its four central features—motivation, goal setting, feedback loops, and self-monitoring—shape decisions about whether to rely on lectures or alternative tools. While curricula often prescribe overarching goals, students also develop personal objectives that drive study behaviors.^11^ Feedback loops and self-monitoring enable adjustment and awareness, but SRL is not innate; early-stage medical students often require support to develop these competencies.^10^ SRL thus would appear to be a useful framework to guide analysis of student motivation and benefits and drawbacks of their chosen resources.

A growing body of research shows that most medical students rely heavily on MED‑COTS and other non‑traditional resources, yet little is known about why these tools are selected and how they shape their learning behaviors and study strategies. When students preferentially use external resources over institution‑developed materials, it raises important questions about the alignment between the formal curriculum and what learners perceive as high‑yield or clinically relevant. This misalignment has implications for faculty time, curriculum design, and the coherence of educational outcomes. Moreover, because these resources influence what students prioritize and retain, their use may ultimately shape clinical reasoning patterns and patient care.

In sum, although prior studies have quantified the prevalence of MED‑COTS use, none have examined the underlying motivations, decision‑making processes, or perceived trade‑offs in depth. Understanding why and how students turn to these resources is essential for informing curriculum development, supporting effective self‑directed learning, and ensuring that educational practices align with the competencies expected in clinical training.

Therefore, the purpose of this study is to explore the use of non-traditional resources in pre-clerkship and clerkship to examine “why and how” students use these resources and perceived advantages and disadvantages compared to traditional resources.

## Methods

### Study Design

We conducted a qualitative research study using one-on-one semi-structured interviews to explore medical student use of non-traditional resources between December 2022 and February 2023. One on one interviews were selected in order to yield more detailed and candid insights than surveys or focus groups by enabling individualized probing and minimizing group bias. A pragmatic research approach informed the study design, reflecting the view that inquiry should focus on understanding how students navigate real world learning contexts. Pragmatism accepts that there may be single or multiple realities, and that actions are inseparable from the situations and contexts in which they occur.^12^^,^^13^ Because students’ resource use behaviors are shaped by socially shared beliefs and worldviews, this approach supported the use of in depth interviews and flexible analytic strategies to generate findings directly relevant to the practical problem under investigation. Ethics approval was obtained from the University of Ottawa.

### Participants and Setting

The University of Ottawa is a 4-year program with the first two years involving primarily classroom instruction (pre-clerkship) followed by two years of rotations in various clinical setting (clerkship). The pre-clerkship curriculum includes ~25 hours of instruction weekly, with a mix of mandatory and optional lectures. Clerkship involves 6-week rotations and comprehensive exams. Pre-clerkship examinations occur on a block basis, typically every 8-12 weeks. Clerkship involves comprehensive exams every 2 blocks (12 weeks). To advance, students must pass all examinations with a minimum score of 60%.

The University of Ottawa is unique in that it offers both an English stream (approximately 118 students) and a French stream (approximately 48 students), where all instruction is in either English or French respectively, following the same curriculum and objectives.

Participants included any students from 2nd or 3rd year who were not part of the research team.  Participants were recruited via the local medical student society's email and social media. Emails were sent to all second- and third-year students (n = ~332) twice over a 2-month period, and social media invitations were posted via the student association twice during this time frame. In addition, 57 students who had previously completed an online survey on a similar topic were subsequently invited to also complete the screening survey.^5^

In order to purposefully sample for medical student year, and high vs low Med-COTS users a short screening survey was administered to any potential participants responding to the recruitment notice.(Appendix A).  We wanted to ensure sampling from both pre-clerkship and clerkship students as they might demonstrate different motivations for learning when moving from primarily class-based learning to learning in the workplace. We also wanted to reduce bias by selecting students reporting low use, as we anticipated more interest from high-end users.

A total of 29 students completed the screening survey and all interviewed. Participants who initially reported not using non‑traditional resources were interviewed first; however, interviews revealed that all had used them, as they did not classify locally developed question banks, flashcards, or note decks as non‑traditional.

Ethics approval was granted by the University of Ottawa Research Ethics Board [H-02–22-7616] and the Vice Dean — Undergraduate Medicine in the Faculty of Medicine. The Declaration of Helsinki principles were followed. Participation was voluntary and all provided informed consent.  Participants were provided with a $25 Amazon gift card for completing the study. There were no interview dropouts or partial interviews.

### Data Collection and Analysis

The interview guide was developed by DM, revised based on feedback from all co-authors, and was informed by the literature1, SRL10, and prior survey data.^5^ The survey gathered demographics, USMLE interest, lecture attendance, resource use, study approaches, and satisfaction (see Appendix B).Medical students in Canada have mandatory national examinations including the Medical Council of Canada Qualifying Exam but are not required to write the USMLE, with a prior survey^5^ demonstrating only 12% of Canadian students at the University of Ottawa were planning on taking the USMLE, with 32% undecided. Since many Med-COTS resources are designed for USMLE preparation this was included in the questionnaire.The interview guide was piloted with three 4th year medical students prior to utilization for clarity. Clerkship students answered additional questions relevant to their training stage.

Interviews were conducted in English by student authors (DM, JM, ML) who did not interview peers from their own cohorts or friend groups to minimize power dynamics. The interviews were held virtually at the time of the interviewees choosing (avoiding proximity to exams) and audio recorded and transcribed by the interviewer using the automatic Microsoft Teams transcripts with clarifications added from field notes. All documents were anonymized prior to analysis, and confidentiality was maintained. These occurred from December 2022 to February 2023, Anonymized transcripts were analysed using reflexive thematic analysis,^14^ combining deductive analysis from SRL theory to examine student motivation and inductive analysis for other themes.^15^ Two authors (DM and ML or JM) independently read the transcripts to familiarize themselves with the data and developed initial codes. All authors applied initial codes to parts of the dataset to help refine codes. Through multiple team meetings, codes were modified with disagreements addressed via consensus through discussion. Final codes were subsequently applied to the data set by the authors (DM, ML, JM). In an iterative fashion, initial themes were developed and refined using a constant comparative approach through several team meetings.  Coding was facilitated using NVivo 12.^16^ Since thematic sufficiency was reached, no further recruitment took place.^12^

### Reflexivity and Trustworthiness

At the time of data analysis, the research team consisted of medical students in pre-clerkship (JM,ML) and clerkship (MD,AK). The students represented the full spectrum of use of non-traditional resources from minimal use (ML), moderate use (JM), heavy use (AK) to very heavy use (DM), thus bringing in different lenses to the dataset. It is noteworthy that as seen in the next section (results), our research team was representative of the three archetypes, lending credibility to the results. As a clinician with over 20 years of experience teaching at the medical school SHM brings in the faculty perspective, and one of a physician who trained when such resources were not available. SHM also has extensive experience in education research. During discussions all members of the research team were encouraged to share their personal beliefs and unique perspectives openly.

## Results

A total of 29 students (18 pre-clerkship students and 11 clerkship students) were interviewed. The average interview lasted 34 minutes (range: 18-65 minutes). Nine participants identified as male and 20 as female, and the majority (n=27) were from the English stream. Participants ranged in age from 21 years to over 30 years: 21–25 (n=26), 26–30 (n=2), and over 30 (n=1). Participant USMLE interest, lecture attendance patterns, and types of resource usage are shown in Table 1.

After defining non-traditional resources for all interview participants, 100% of students (n=29) described using some form of non-traditional resources, despite 8 (5 pre-clerkship students and 3 clerkship students) having stated otherwise in the pre-interview screening survey. Descriptions of the top 5 resources for both locally student-made and MedED-COTS resources can be found in Table 2.

We generated four themes from our participant interviews. The first two address the “why” or motivations for using non-traditional resources, the third addressing goal setting and the fourth addressing “how” students engage with resources.

### Traditional Curriculum - Repetitive and Inflexible

Many participants suggested current traditional offerings did not meet their needs. Students frequently voiced that attending live lectures did not add additional information beyond that obtained from slide decks or pre-existing student note sets, and that these notes could replace lectures.

“A lot of lectures aren’t effective.” [P03 clerkship student]

Not all students, however, were dismissive of lectures. Some noted that live lectures worked better for their learning preferences due to their auditory component “I retain the information better that way.” [P01 pre-clerkship student]. Some students valued the role of lecturers in the curriculum because they could impart “clinical pearls” [P04 clerkship student] or answer questions, while others noted that they liked the structured, scheduled nature of regular lectures.

**Table 1:** Demographics: Self-reported Lecture attendance, time and resource selection, USMLE interest*

	Response	Total number ofstudents (n=29)	Total numberof pre-clerkshipstudents(n=18)	Total numberof clerkshipstudents (n=11)
USMLE Interest	Committed to taking the USMLE exam in the future	1	0	1
	Considering taking USMLE in the future	5	4	1
	No interest in USMLE	23	14	9
Lecture Attendance	Attend all/most lectures	12	8	4
	Attend some lectures	8	7	1
	Attend few/no lectures	9	3	6
Non-TraditionalResource Usage	Heavy Utilization^**^	9	4	5
	Balance of Non-Traditional and Traditional	15	9	6
	Little utilization	3	3	0
	Uncertain	2	2	0
Non-TraditionalResource Selection	Only locally student-maderesources	10	9	1
	Mixture of local student-made and MedED-COTS resources	18	9	9
	Only MedED-COTS resources	1	0	1

^*^Responses are categorized based on participant’s free text response during the interview. ^**^Heavy utilization was noted as stating that they predominantly use MedED-COTS resources.

Timing of lectures was highlighted. Students felt that the pace of lectures were “too slow” [P19 clerkship student] and some addressed this by adopting a strategy of watching recorded lectures at faster speeds on their own time. Despite this, when asked about the removal of lectures, most supported the continued existence of lectures, “I feel like it’s really helpful.” [P12 pre-clerkship student] while some suggested that live lectures could be replaced by recorded lectures or self-learning modules.

Of note is that some students chose whether to attend non-mandatory lectures based on scheduling factors (mandatory lectures on the same day) with a few students indicating they appreciated the social aspect of lectures.

“Nowadays I pretty much go to lecture for like the social aspect of it.” [P13 pre-clerkship student]

### Non-Traditional Resources - High yield and Flexible

The most common reasons for choosing non-traditional resources were time efficiency and flexibility. Most students agreed that their resource usage lessened their study time, while also allowing more scheduling flexibility.

“I could do so much more with my time compared to if I was relying only on the faculty-approved things.” [P20 clerkship student] 

Clerkship students reported more reliance on non-traditional resources, noting that this was in large part due to a lack of traditional resources.

“I think like clerkship-wise I feel more comfortable with the external resources.” [P02 clerkship student]

Generally, resources were viewed as more trustworthy if there was heavy utilization by many of their peers, and iterative revision with citations by experts were included.  Interestingly, some students had not given much thought to accuracy.

The idea of non-traditional resources being high quality was noted repeatedly. Some students reported that they find the extra detail in non-traditional resources helpful.

 “They’re gold standards, they’ve been proven time and time again to give you the right, […] the good amount of information that is required to pass your exams, while also still being good on wards.” [P19 clerkship student]

**Table 2:** Most commonly used non-traditional (student and MedED-COTS) resources and descriptions mentioned in interviews. Prices from 2024 for 1 year of comprehensive access and do not reflect discounts.

Resource	Type ofresource	Number ofusers (n=29)n (%)	Description	Cost per year($ USD 2024)	Perceptions of our participants
Upper Year Notes	Local Student Made	22 (75.9)	Notes from previouslectures transcribed and enhanced by priorstudents	Free	Good for replacing lectures, however, can be excessively detailed and no references.
Anki Decks	Local andExternalStudent Made	17 (58.6)	Spaced repetition flashcards created either locally or by other medicalstudents	Free	Good for consistent studying (avoiding cramming) and excellent memorization but technically difficult to setup and cards can build up.
Upper Year Flow Charts	Local Student Made	16 (55.2)	Graphical representations of key lecture components made by prior students.	Free	Well organized and concise, can be too concise.
Student Question Bank	Local Student Made	16 (55.2)	An organized project to build student created questions for all lecture topics	$108($20 at time of study)	Good for testing understanding but has poor formatting.
Amboss	Commercial	16 (55.2)	Clinical knowledge library and question bank	$479(with question bank)	Constantly updated and easy to search, question banks are useful, good for summarizing and highlighting key concepts, The Anki addon is appreciated.
Student Mentoring Centre Slides	Local Student Made	13 (44.8)	Slide decks of “High yield” concepts created by prior students	Free	Good for big picture concepts but sometimes “too concise”
Osmosis	Commercial	10 (34.5)	Clinical knowledge videos and question bank	$598	Good for short, concise overviews and visuals
UpToDate	Commercial	7 (24.1)	Clinical decision support tool	$319	Used for clinical question solving but sometimes overwhelming and “too advanced” .
Med Bullets	Commercial	3 (10.3)	Question bank	Free to $250	Good for exam performance however American content
Boards& Beyond	Commercial	2 (6.9)	Clinical knowledge videos and question bank	$249	Good for learning about a topicinitially.

Exam performance was also frequently noted to be improved by non-traditional resources, especially student-made resources, in contrast to the traditional curriculum. For some, it was essential.

“Without third party resources I honestly don’t know how I would study.” [P25 pre-clerkship student]

Students identified resources fell into one or multiple of the following categories: 1) knowledge-focused content libraries, either MedED-COTs or student made, 2) question banks (e.g. Ottawa Question Bank, Amboss), and 3) clinical decision-support tools (e.g. UpToDate). These categories were viewed as complementary.

“I learn it from the third-party resources and then after that, the practice questions that I would be doing are from the Student Mentoring Center as well as the weekly quizzes.” [P20 clerkship student]

Of note, a minority of students had a contrasting negative outlook on non-traditional resources, identifying many disadvantages including cost, concerns over accuracy, efficiency, content discrepancies.

“So I do think they [Student Resources] could be very helpful, but I would prefer to purchase something made by a physician or researcher.” [P12 pre-clerkship student]

Peers played a prominent role in recommending resources, as did medical student associations, and web searches. Observation of the utilization of resources used by staff physicians and residents was another reported influence.

“There’s a lot of word of mouth I find with Med students.” [P08 clerkship student]

Notably, the impact of the USMLE interest was negligible, as shown in Table 1, despite many MedED-COTS resources being designed for USMLE preparation.

### Studying for Today’s Exam or Tomorrow’s Patients

Both pre-clerkship students and clerkship students appeared to be broadly identified as falling into two studying mindsets. There was a present, exam-focused mindset which we labelled “Pass Now.” This manifests itself in numerous ways, wherein students’ studying foci are directly informed by their immediate exam focus, as opposed to their long-term clinical performance.

“It’s really just about passing the exams for me right now.” [P13 pre-clerkship student]

In contrast, a smaller contingent of students demonstrated a future-oriented mindset. For them, exam performance is important, but future clinical performance is more important. This is the “Learn for the future” group.

“I would rather know information that’s even more relevant for me down the line.” [P14 pre-clerkship student]

### Utilization Archetypes: The Traditionalist, The Supplementer, The Reformer   

When examining the patterns of resource utilization across the data, students fell into three major archetypal patterns, summarized in Table 3.  These archetypes were most common in pre-clerkship students.  However, clerkship students also conform to these groups with fewer traditionalists due to the lack of a formal curriculum in the clerkship years. The first archetype, “Traditionalists”, are heavily reliant on the traditional curriculum, utilizing almost exclusively student-made lecture notes. The second, “Supplementers”, are the most common archetype with a key feature including attending lectures at least some of the time, while also having heavier reliance on non-traditional resources including MedED-COTS resources. The third category was labelled “Reformers.” Reformers reject lectures as their main modality for learning. Instead, they use non-traditional, non-lecture-based resources. These students generally, though not always, rely heavily on Anki. They may completely eschew official resources and are more likely to express interest in the USMLE.

**Table 3:** Overview of the three major types of resource utilization patterns/archetypes.

“The Traditionalist”​	“The Supplementer”	“The Reformer”​
Attends all or almost all lectures​	Attends at least some lectures	Does not attend any or only rarely attends lectures​
Does not use MedED-COTS resources AND/OR minimal student resources that are lecture note based​	Uses MedED-COTS AND/OR student resources alongside traditional curriculum	Uses combination of MedED-COTS AND/OR student resources instead of traditional curriculum​
*“So I know like everything I need to know is already in [the] lecture. So to me like because lecture already has so much information. If I already kind of like understand what’s going on then I don’t really have the time or effort in my brain to be able to like process like external resources”* [P10 pre-clerkship student]​	*“It is helpful to be there and see what the instructor you know highlights as being important and I think it is good to listen to the lectures and see you know what these specialists speak about… ​…I’ve certainly gotten much more value out of [Amboss] in terms of helping me understand content and having someone show me what’s high yield.”​*[P25​ pre-clerkship student]	*“ I do not attend them is because I find there’s very little insight that the lecturer gives beyond what’s in the PowerPoint slides… …Whereas I found like on Anki and Amboss those resources gave me the important bits that were high yield and made everything more absorbable and less overwhelming.”* [P20​ clerkship student]

## Discussion

In this study, we advanced understanding of medical students’ motivations and use of non-traditional learning resources^1^^,^^5^^,^^6^ by employing an interview-based design that allowed deeper exploration of students’ experiences. We found near-universal utilization of such resources, at levels exceeding those reported in prior studies^3^ with dissatisfaction with the traditional curriculum serving as a central motivator. Peer influence, including both recommendations and resource development by upper-year students, was critical in guiding resource choice. These findings echo those of Lawrence,^6^ while extending the literature by identifying three distinct learner archetypes that provide a useful framework for conceptualizing resource use. We also found that students’ orientation—whether future- or present-focused—shaped resource selection, particularly in contexts where pass/fail curricula shifted attention away from exam performance.

Students identified several perceived shortcomings of the traditional curriculum, noting that lectures were inefficient, of limited relevance, and inconveniently scheduled. Scheduling has been previously shown to affect lecture attendance,^17^ as has the use of accelerated recordings. Some students acknowledged attending lectures primarily to ensure exposure to exam-relevant material, while others sought alternative or supplementary non-traditional resources, highlighting the motivational component of SRL. Still, students recognized enduring benefits of lectures, particularly the transmission of “clinical pearls” and the value of faculty expertise, leading to reluctance to eliminate lectures altogether.^18^

Consistent with prior studies^4–6^ students in our study relied heavily on peer recommendations when selecting resources. Efficiency, relevance, and formatting were central to their choices, with accessibility and comprehensiveness being double-edged factors that could facilitate or overwhelm learning.^4^^,^^19^ Notably, students in our Canadian context, who had little interest in writing the USMLE, reported less frequent use of resources that are highly popular in the United States.^20^

Few students critically evaluated the quality or trustworthiness of non-traditional resources, highlighting a concerning gap in the monitoring component of SRL. Prior research found that one-third of students did not question the quality of medical apps, with fewer than 20% seeking peer ratings and only 12% checking against textbooks or lectures.4 In our study, some students appeared to validate resources by their alignment with short-term exam goals (“pass now”), while others prioritized clinical relevance (“study for the future”). These findings raise important concerns for faculty, as gaps in appraisal of resources may ultimately influence the quality of future patient care.

Our identification of three new archetypes—Reformers, Traditionalists, and Supplementers—adds nuance to existing literature. Reformers, who rarely attend class, rely almost exclusively on non-traditional resources, while Traditionalists, who prioritize lectures, view the curriculum as their central structure. Supplementers, positioned between these extremes, may be especially valuable in identifying gaps in the official curriculum. Prior literature described non-traditional resources as a “parallel curriculum” for USMLE preparation,^21^ but our findings suggest a “supplemental curriculum,” where students strategically combine traditional and non-traditional tools. This distinction advances prior work,^6^^,^^21^ demonstrating that many students see value in institutional curricula while still seeking the efficiency and convenience of external resources. Importantly, even when institutions integrate external resources into curricula, students often continue to use their own preferred materials.^22^

The archetypes, coupled with the dichotomy of present- versus future-oriented studying, map directly onto SRL constructs of motivation and goal setting. While all students ultimately aim to pass exams and develop clinical competence, their self-generated goals differ. Pre-clerkship students tended to view exams as the endpoint, relying heavily on peer-developed “high yield” resources to avoid “overstudying,” consistent with prior reports.^6^ In contrast, clerkship students were more likely to situate their studying in clinical contexts, recognizing exams as necessary but secondary to the development of practice-relevant knowledge. These differences illustrate how SRL manifests in distinct study strategies and priorities.

SRL is increasingly recognized as an essential skill for lifelong learning. Faculty may benefit from reframing student non-attendance at lectures—often criticized or considered unprofessional^23^—as a potential marker of SRL engagement, particularly among Reformers who take an active role in directing their own learning. Our findings also highlight the complementary use of multiple non-traditional resources, such as combining question banks with content libraries, which was common across student archetypes. While multiple-resource use has been reported previously,^6^ our study adds insight into how students combine tools to engage in clinical reasoning and knowledge consolidation. The widespread use of question banks, even among students not preparing for the USMLE, illustrates how learners actively use feedback loops to identify gaps and monitor progress—key features of SRL.

A key strength of this study is the composition of the research team, which included four medical students representing both pre‑clerkship and clerkship stages, diverse levels of engagement with non‑traditional resources, and both English and French program streams. This diversity enhanced the study’s reflexivity and ensured that multiple learner perspectives informed data collection and interpretation. The major limitation of this study is its focus on a single Canadian medical school with a lecture‑ and small‑group–based curriculum, which may limit transferability to other contexts. As with all qualitative research, the aim is not generalizability but rather to provide rich, contextualized insights that may be informative to similar settings.

Self-selection is also a potential limitation, and while we did attempt to target individuals reporting no utilization of non-traditional resources, we found all students utilized these resources to some degree. While offered in French, no French stream participants ultimately choose to interview in French as well. The use of student interviewers also serves as a potential limitation although these interviewers purposely avoided interviewing their peers and friends. Additionally, for privacy and ethical reasons, linkage between this interview-based study and prior survey work on the same population was not possible.

## Conclusions

Four themes emerged: two reflecting students’ motivations for turning to non‑traditional resources—the perception that the traditional curriculum is repetitive and inflexible, contrasted with the high‑yield flexibility of external tools; one highlighting differences in goal setting, with students studying either for today’s exam or tomorrow’s patient; and one illustrating how learners engage with resources through three distinct archetypes—the Traditionalist, the Supplementer, and the Reformer.

Taken together, we suggest that these findings point to several practical, potential steps for medical educators. Addressing curricular rigidity by streamlining lecture content, increasing flexibility in delivery, auditing scheduling practices, and co‑designing improvements with students may help reduce the inefficiencies that drive learners toward external tools. At the same time, institutions can better align with student practices by formally curating and vetting high‑yield non‑traditional resources, embedding selected tools within the curriculum, supporting faculty in their use, and developing partnerships to ensure equitable access. Strengthening students’ self‑regulated learning skills is also essential, including explicit instruction in goal setting, monitoring, and resource appraisal, as well as guidance to balance short‑term exam needs with long‑term clinical competence—particularly in pass/fail environments. Leveraging the three learner archetypes can further inform curriculum planning by recognizing diverse learning approaches, engaging Supplementers as indicators of curricular gaps, providing Traditionalists with structured pathways that incorporate vetted external resources, and viewing Reformers’ non‑attendance not as disengagement but as an expression of SRL, warranting supports that meet them where they are. Further, particularly more international, qualitative work, to explore resource utilization in other non-American settings, particularly in lower-resource settings as many of these resources are Internet and mobile device based, would further build on this and explore opportunities for curriculum development.

### Acknowledgments

The authors wish to thank the Aesculapian Society 2022-2023 executive, for aiding the distribution of the screening survey. Thank you to the fourth-year students who reviewed the interview guide.

Funding: This work was supported by an Ontario Medical Students Association (OMSA) Medical Student Educational Research Grant (MSERG).

### Conflict of Interest

The authors declare that there is no conflict of interest.

## Supplementary material

Supplementary file 1 Appendix A. Recruitment Screening Questionnaire

Supplementary file 2 Appendix B. Interview Guide
