# Supplementary material for: Traditionalist, supplementer, or reformer? medical student use of non-official curricular resources
Source: Int J Med Educ. 2026 Jul 3;17:97–108. doi: 10.5116/ijme.6a31.72ef (PMC13577449; doi:10.5116/ijme.6a31.72ef)
Supplement: Supplementary file 1 [file ijme-17-97-S1.pdf]

Appendix A

Recruitment Screening Questionnaire

Qualitative Interview

Commercial and third-party resource usage in undergraduate medical education - recruitment

Thank you for taking the time to participate in this study!

Title of study: Commercial and Community Third-Party Extra -Curricular Resource Usage in Pre-clerkship Undergraduate Medical Education

Name of Principal Investigator: Donovan Makus

Affiliation: University of Ottawa Faculty of Medicine

Email address: Donovan.Makus@uottawa.ca

Invitation to Participate: You are invited to participate in the abovementioned research study conducted by Donovan Makus, Anshu Kashyap, Mark Labib, and Dr. Susan Humphrey-Murto. You are being asked to complete a brief (<5 minute) screening survey to be selected to participate in a ~30-minute confidential interview with a member of the research team.

Purpose of study: This study aims to better understand the usage of extracurricular and commercial resources by preclerkship medical students. We are seeking your input regarding your use of curricular resources outside the formal medical school curriculum, and the impact COVID-19 has had on your studies.

Participation: You are being asked to complete a brief online survey and participate in a ~30-minute online interview using Microsoft Teams ® or Zoom ® if you are selected for an interview. This interview will be audio recorded to generate a transcript of the interview for later analysis. You may refuse or decline to answer any question or leave the interview at any time without fear of reprisal or ill treatment.

Risks: Your participation in this study will entail no identifiable risk as you are sharing your individual study methods and experiences. You may withdraw from this study at anytime, without requiring a reason, by contacting the principal investigator.

Benefits: Your participation in this study will improve understanding of preclerkship medical student studying patterns and extracurricular resource usage. The information should be helpful to understanding the gaps in the formal curriculum.

Confidentiality and Privacy: The information you will share will remain strictly confidential. The contents will be used only for the purposes of academic medical education research and that your identity will be protected. All data will be coded with an independent study number so as to maintain anonymity, and any data presented will be in an aggregated format. Thus, you will not be identifiable in any publication or presentations resulting from this study. Only members of the research team working at the University of Ottawa may review the full study data records for audit purposes.

In order to minimize the risk of security breaches and to help ensure my confidentiality, it is recommended that you use standard safety measures, such as signing out of my account, closing my browser, and locking my device when you are no longer using it/when you have completed the study.
You may withdraw from the study and request the deletion of your study data at any time without requiring a reason.

Conservation of Data: The data collected (survey datasets) will be securely stored in uOttawa Single Sign On protected SurveyMonkey and OneDrive accounts that are only accessible to members of the research team. Any analysis requiring downloading of data will be completed on password protected encrypted personal computers over secure networks. Data will be stored for 5 years following the completion of the study. Interview recordings will be used solely to generate and confirm the accuracy of the transcribed transcript. The audio recordings will be stored in a secure location and listened to only by members of the research team.

Funding: This project is generously supported by an Ontario Medical Students Association (OMSA) Medical Student Education Research Grants (MSERG).

Compensation: To thank you for your contribution to the research project, you will receive a $25 Canadian dollar gift card to Amazon.ca if you are selected to participate in the interview. The gift card must be accepted as awarded or forfeited and cannot be redeemed for cash. Your name and the contact information you have provided will be kept confidential and then destroyed once the gift cards have been awarded and the study data collection complete. If you choose to withdraw from the study after being selected for an interview, you will still receive this compensation.

If you have any questions about the study, you may contact the researcher or their supervisor. If you have any questions regarding the ethical conduct of this study, you may contact the Office of Research Ethics and Integrity via email (ethics@uottawa.ca) or telephone (613-562-5387).

It is recommended that you save a copy of this consent form for your records.

Acceptance: By completing and returning the survey, I am consenting to participate in the collection of my responses for the purpose of screening my eligibility to participate in an interview.

* 1. What is your name?
* 2. What is your cohort?
MD2025 (MS2)
MD2024 (MS3)
MD/PhD (MS1)
MD/PhD (MS2)
MD/PhD (PhD phase)
* 3. Please Identify your language stream
French
English
* 4. Are you available to complete a ~30-minute virtual interview in December or
January/ February
Yes
No
None of the above
* 5. What email address would you like us to use to contact you?
* 6. Do you use commercial or third-party resources? (E.g., Anki, Amboss)
Yes
No
Thank you for completing the survey. We will contact you if you are selected for an interview.
