# Supplementary material for: Traditionalist, supplementer, or reformer? medical student use of non-official curricular resources
Source: Int J Med Educ. 2026 Jul 3;17:97–108. doi: 10.5116/ijme.6a31.72ef (PMC13577449; doi:10.5116/ijme.6a31.72ef)
Supplement: Supplementary file 2 [file ijme-17-97-S2.pdf]

Appendix B

Interview Guide

Interviewer Guide English

Thanks for taking the time to meet with me. We are interested in better understanding the way medical students study in preclerkship and use resources that are not part of the official curriculum. So the plan for this interview is to explore the things that you think are important in your approach to studying in medical school, your approach to lectures, use of external resources and some brief biographical questions.

Our scientific team is interested in general lessons about your experiences, so feel completely free to be open and honest about your views. We do not want you to try to give us the "right answer," we want to hear your open and honest view. No released information will ever be able to identify you or anyone you talk about. Reports stemming from this work will only use unidentifiable quotes or describe the discussions we have in aggregate form. I have a series of questions here, and I may ask for some points of clarification throughout just to make sure I understand your thought process, but feel free to mention whatever you feel is relevant at any time.

Any questions before we start? Is it alright if I record our conversation so that I can review it later?

Demographic questions

- What is your projected graduation year and stream (**Prompt:** French, English, CNFS, MD/PhD)?
- What best describes your gender identity? (**Prompt**: female, male, self-describe if comfortable, decline to state)
- Which age category do you fall into? (**Prompt:** <21, 21-25, 26-30, >30, prefer not to indicate)
- What is your academic background prior to medical school? (**Prompt**: Undergraduate, postgraduate, field, any gaps in time spent in school prior to med school)
    - i. **Follow-up:** Has this affected your approach to medical school studying? If so, how?

USMLE & Lecture Attendance

1. How do you think preparing for future national exams impacts the way in which you study and the resources you use (if at all)? (**Prompt:** USMLE Step 1 and MCCQE)
2. Do you attend non-mandatory lectures? Please explain why you do or do not attend.
    - i. **Follow-up:** Would you support the removal of lectures if they were replaced with some alternative? If so, what alternative would you prefer.
3. Do you use recordings of lectures? Please explain why or why not?
    - a. **Follow-Up**: Has your use changed over time? If so, why?

Resources and Approach to Studying

Only for MD2024 cohort: Where applicable, for these questions I'd like you to think of both your pre-clerkship experience and your clerkship experience when answering. [Prompt: **Ask them about both their approach in clerkship and preclerkship if they do not spontaneously mention it for 4., 5.,7., and 8.).**

4. Tell me about the resources you use that are not part of the formal curriculum. What formal curriculum resources do you use? Where did you learn about these resources?
    - a. **Follow up** – what do you perceive as the advantages and disadvantages?
        - i. If Anki not mentioned ask if tried, If used, which decks, +/-s of the deck? How do they unsuspend cards?
        - ii. ASK ABOUT QUESTION BANKS
        - **iii. If not mentioned in disadvantages ask:** Do you feel that unofficial resources cover the same content covered by official resources or are there discrepancies?
    - b. **Follow-up if in French stream:** How much of your resource usage is in French and how much is in English?
        - i. **Follow-up:** How does this affect your studying and preparation for tests?

   c. **Follow-up**: Do you think your future career aspirations impact how you study and the resources you use (Prompt: for example if you plan on being a family doctor vs a specialist).
5. Are there certain areas of the curriculum (lectures, practical, SIM, CBL) you preferentially use these resources to study? Can you elaborate on reasons for this
   a. **Follow-up:** Can you describe your approach to using official and extra-official resources to study for physician skills development (PSD)?
   b. **Follow-up:** Where do you believe the balance lies between practicing physician skills and relying on other forms of study such as reading or memorizing?
6. **Only for the MD2024 cohort:** How well prepared did you feel for clerkship? Do you think the formal curriculum was more effective than external resources in preparing you?
   a. Has your use of external resources changed since you entered clerkship?
   b. Can you describe how the transition to clinical environments affected your approach to learning?
      i. How important do you think knowledge is to success in clerkship?
7. If you purchased third-party resources, was it worth the money? Why or why not?
   a. **Follow-up:** How much would you be willing to spend?
8. Some third-party resources are created by medical students, not researchers or physicians. Do you view these resources differently?
   a. **Follow-up:** How do you determine you can trust the content of the resources you're using? Why or why not?

Study Time

9. Now thinking about the time you spend studying (individual, lectures, group).
   a. **Follow-up**: How does this change before and after tests?
   b. **Follow-up:** How much time is spent using official uOttawa content in comparison to third-party content?
   c. **Follow-up:** Has your approach to how much time you spend studying changed over time?
10. Do you do any studying in the summers? If so, please describe.
    a. **Prompt:** Keeping up with Anki, structured review, question banks, Twitter, etc...
11. Do you find that third-party resources are less or more time efficient to your learning than the official curriculum?

Studying Method Satisfaction

12. How satisfied are you with your current method of studying and use of resources both official and unofficial?
    a. **Follow-up**: Does the use of third-party resources affect your satisfaction?
13. Like many medical schools the University of Ottawa has a pass/fail curriculum grading approach. How does this affect your approach to studying?
    a. Where do you believe the balance lies between studying for future clinical practice, in clerkship or beyond, vs. studying for exams now?
14. You are given an unlimited budget and complete free rein to restructure your medical school's pre-clerkship curriculum in any way you want. Please describe what your ideal pre-clerkship curriculum would look like.
    a. **Follow-up**: What is the role of lecturers/staff physicians in this curriculum?
    b. **Follow-up**: What is the role of medical students in this curriculum?

Before we conclude, are there any comments you made you would like to revisit or change? If not, why did you decide to participate in this research study?
Thanks for spending the time talking to me today.

Field notes:

Note participant engagement and general impressions immediately post-int
